# Dysregulation of Phosphoinositide 5-Phosphatases and Phosphoinositides in Alzheimer's Disease

**DOI:** 10.3389/fnins.2021.614855

**Published:** 2021-02-25

**Authors:** Kunie Ando, Christophe Erneux, Mégane Homa, Sarah Houben, Marie-Ange de Fisenne, Jean-Pierre Brion, Karelle Leroy

**Affiliations:** ^1^Laboratory of Histology, Neuroanatomy and Neuropathology, Faculty of Medicine, Université Libre de Bruxelles Neuroscience Institute, Université Libre de Bruxelles, Brussels, Belgium; ^2^Institute of Interdisciplinary Research in Human and Molecular Biology (IRIBHM), Campus Erasme, Université Libre de Bruxelles, Brussels, Belgium

**Keywords:** synaptojanin, microglia, SHIP2, Alzheimer's disease, phosphoinositide phosphatases, phosphoinositides

## Introduction

Alzheimer's disease (AD) is the most common type of dementia and its prevalence is expected to rise in response to an aging human population. Yet, there is no disease-modifying drug currently available. The neuropathological hallmarks of AD are amyloid plaques composed of amyloid ß (Aß) peptides derived from successive cleavages of Amyloid Precursor Protein (APP) and neurofibrillary tangles (NFTs) constituted of the microtubule-associated protein tau (Brion, 2006). In AD brains, tau is hyperphosphorylated and aggregated to form paired helical filaments (PHF-tau). The disease pathogenesis precedes the overt clinical symptoms by 10–15 years. Early diagnosis and biomarkers are thus crucial for future clinical trials of AD. However, current standard biomarkers such as amyloid-PET scans are highly expensive and the patients are exposed to a considerable amount of ionizing radiation at each test. Cerebrospinal-fluid analyses for Aß and tau are highly invasive due to lumbar punctures (Dolgin, 2018). We need to search for additional biomarkers that are less expensive and less invasive.

**Figure 1:**
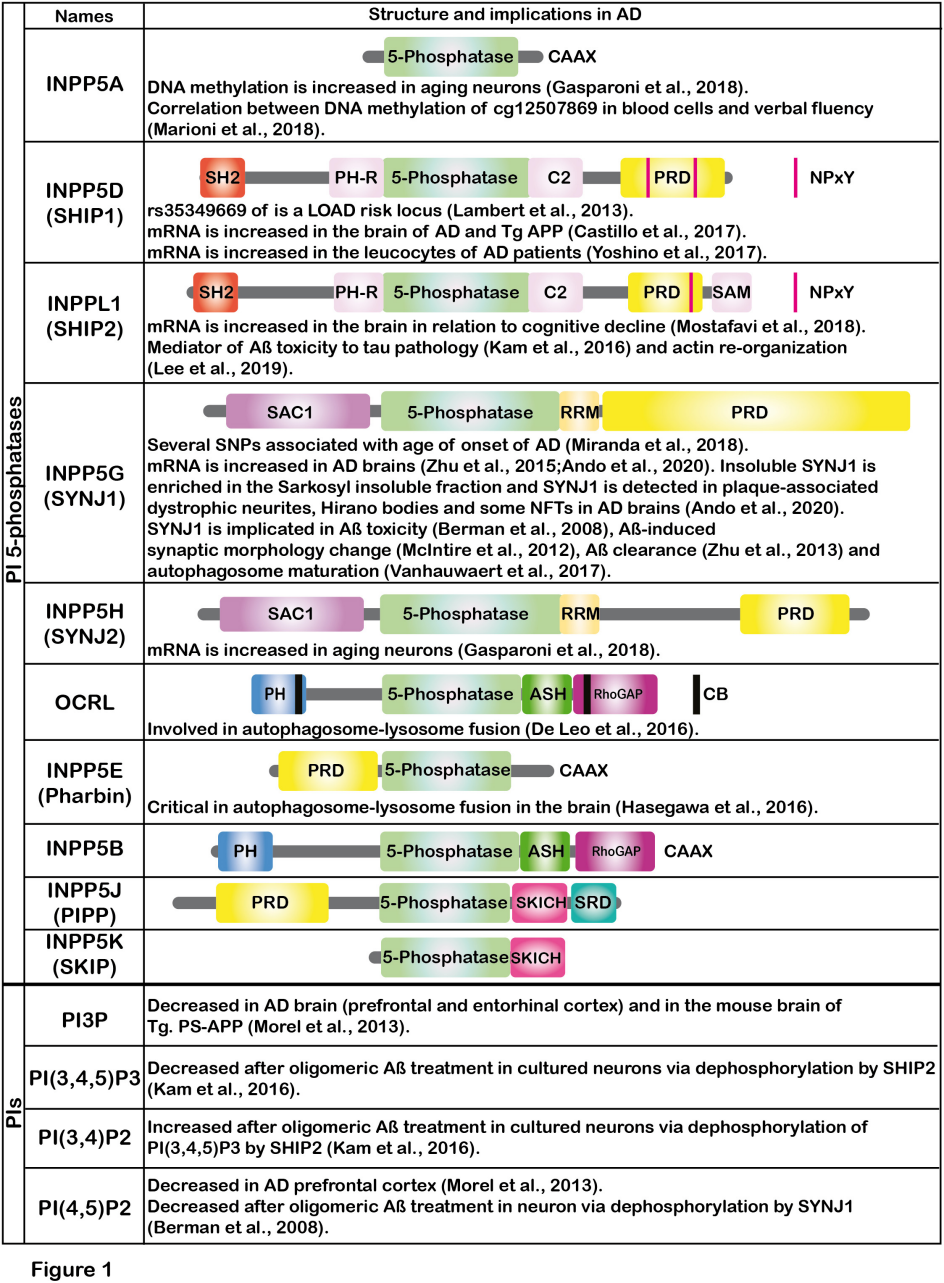
The figure shows the schematic illustrations of the major domains of PI 5-phosphatases and summarizes the implications of PI 5-phosphatases and PIs in AD. Each PI 5-phosphatase contains a highly conserved 5-phosphatase domain shown in green. PI, phosphoinositide; SHIP1, SH2 domain-containing inositol polyphosphate 5-phosphatase-1; SHIP2, SH2 domain-containing inositol polyphosphate 5-phosphatase-2; SYNJ1, Synaptojanin 1; SYNJ2, Synaptojanin 2; OCRL, oculocerebrorenal syndrome of Lowe, PIPP, proline-rich inositol polyphosphate 5-phosphatase; SKIP, skeletal muscle and kidney enriched inositol phosphatase; CAAX, CAAX motif; SH2, Src homology 2; PH, Pleckstrin-homology; PRD, proline-rich domain; NPxY, a conserved tyrosine phosphorylation motif (Asn-Pro-x-Tyr) for binding to a phospho-tyrosine binding (PTB) domain; SAM, sterile alpha motif; SAC1, suppressor of actin 1; RRM, RNA recognition motif; ASH, ASPM-SPD2-Hydin domain; RhoGAP, Rho GTPase-activating protein domain; CB, clathrin binding domain; SRD, serine rich domain; SKICH, SKIP COOH terminal homology domain.

Emerging evidence suggests that Aß modifies the metabolism of phosphoinositides (PIs) (Berman et al., 2008; Kam et al., 2016). PIs control major signaling pathways and cell processes in eukaryotic cells. Ten enzymes of the inositol and phosphoinositide 5-phosphatases (hereafter, PI 5-phosphatases) have been identified in the human genome i.e., INPP5A, INPP5D (SHIP1), INPPL1 (SHIP2), INPP5G (SYNJ1), INPP5H (SYNJ2), OCRL, INPP5E (Pharbin), INPP5B, INPP5J (PIPP) and INPP5K (SKIP) (Figure 1). Except for INPP5A, PI 5-phosphatases essentially dephosphorylate PI(4,5)P2 and PI(3,4,5)P3 at the 5-position of the inositol ring with different degrees of catalytic efficiency and selectivity for each isoenzyme. PI 5-phosphatases are involved in fine-tuning regulation of PI(4,5)P2 and PI(3,4,5)P3, key intracellular signaling molecules known to be present in different subcellular compartments of the cells. Recent genetic and epigenetic studies have unequivocally suggested that some of the PI 5-phosphatases are implicated in AD, in addition to several other human diseases (Ramos et al., 2019). In this opinion article, we review recent findings on the PI 5-phosphatases in relation to AD, aging and cognitive functions. Such information could be potentially useful for developing novel biomarkers for AD in the future.

### INPP5A and Cognitive Functions

Unlike other PI 5-phosphatase family members, INPP5A recognizes only soluble inositol 1,4,5-trisphosphate [Ins(1,4,5)P3] and inositol 1,3,4,5-tetrakisphosphate [Ins(1,3,4,5)P4] as substrates. INPP5A is ubiquitously expressed including in the hippocampus and prefrontal cortex, the brain regions highly affected in AD, and is abundantly detected in cerebellum (Liu et al., 2020). INPP5A negatively controls the mobilization of intracellular calcium by decreasing Ins(1,4,5)P3 levels (De Smedt et al., 1997). DNA methylation of the *INPP5A* gene is increased in association with aging in neurons (Gasparoni et al., 2018). Meta-analysis of blood-based DNA methylation has shown that the methylation of cg12507869 located in the *INPP5A* gene had a significant negative correlation with phonemic verbal fluency and was associated with logical memory and vocabulary (Marioni et al., 2018). Blood-based DNA methylation of cg12507869 in the *INPP5A* could be thus considered as a potential biomarker for aging and cognitive functions.

### SHIP1 and AD

SHIP1 is a hematopoietic-specific PI 5-phosphatase activated downstream of a multitude of receptors for growth factors, cytokines, antigens, immunoglobulin and toll-like receptor agonists. Once activated and correctly localized, SHIP1 generally acts as a negative regulator of signaling processes in hematopoietic cells, for example on the B cell receptor activation signaling pathway (Ramos et al., 2019). SHIP1 is detected in the brain, primarily in microglia reflecting its myeloid origin. Genome-wide association studies (GWAS) have identified the risk variant rs35349669 in *INPP5D*, the gene encoding human SHIP1 for late-onset AD (Lambert et al., 2013). *INPP5D* mRNA is significantly upregulated in human AD brains and in transgenic mouse brains with knock-in mutations of APP^NL−G−F/NL−G−F^ (Castillo et al., 2017). *INPP5D* mRNA expression in peripheral leucocytes is elevated in early AD but is decreased with cognitive decline (Yoshino et al., 2017). Further long-time follow-up of the participants would be necessary to decipher the correlation between the level of *INPP5D* mRNA and cognitive decline. Since SHIP1 converts PI(3,4,5)P3 to PI(3,4)P2, the amounts of these PIs in the blood leucocytes may also be altered and needs to be further investigated (as discussed in section PI Metabolism and Autophagic-Endosomal-Lysosomal Abnormalities). Taken together, the level of *INPP5D* mRNA in leucocytes could be an interesting target to develop a blood-based biomarker in the early stages of AD.

### SHIP2 and AD

SHIP2, encoded by *INPPL1*, is ubiquitously expressed including in the brain (Muraille et al., 1999). By using PI(3,4,5)P3 as substrate, SHIP2 controls PI(3,4)P2 content, a major SHIP2 product (Ghosh et al., 2018). SHIP2 can also dephosphorylate PI(4,5)P2, another albeit less potent substrate (Elong Edimo et al., 2016). PI(3,4)P2 is scarce under normal conditions but increases through signaling following PI 3-kinase activation. This lipid plays critical roles as a second messenger in cell migration, polarity, feedback control of PI(3,4,5)P3 generation, and basal mTORC1 activity (Ramos et al., 2019). SHIP2 is directly implicated in several human diseases: mutations in *INPPL1* cause opsismodysplasia, a rare autosomal recessive disease characterized by delayed bone maturation (Fradet and Fitzgerald, 2017). SHIP2 is also upregulated in some cancer cells, particularly in aggressive human breast cancer cells (Ghosh et al., 2018). SHIP2 negatively regulates insulin/IGF-I actions and is implicated in type 2 diabetes and metabolic syndrome (Marion et al., 2002). Recent network-based approach has unraveled that SHIP2 is also linked to AD and cognitive decline: upregulation of *INPPL1* transcript in the brain significantly correlates with cognitive decline in human AD patients (Mostafavi et al., 2018). The same study also reported that SHIP2 immunoreactivity was detected in astrocytes and neurons in the *post-mortem* human brain tissues of AD patients and that lentivirus-mediated down regulation of SHIP2 in cultured astrocytes significantly reduced Aß production (Mostafavi et al., 2018). Other independent studies have reported SHIP2 functions as a mediator of amyloid toxicity via tau hyperphosphorylation (Kam et al., 2016) and actin-cytoskeleton reorganization (Lee et al., 2019). Kam *et al*. reported that the interaction between Aß and the FcγRIIb immuno-receptor leads to a translocation of SHIP2 to the plasma membrane to form a protein complex in which SHIP2 dephosphorylates PI(3,4,5)P3 into PI(3,4)P2. Increased amounts of PI(3,4)P2 lead to decreased inhibitory phosphorylation of GSK3ß at Ser9 *via* endoplasmic reticulum (ER) stress in cultured neurons (Kam et al., 2016). Consequently, tau phosphorylation by GSK3ß is increased by Aß *via* FcγRIIb-SHIP2 complex (Kam et al., 2016). SHIP2 inhibitors are thus under active scrutiny as a novel therapeutic target for AD. Actually, SHIP2 inhibitors represent new treatments for several diseases: SHIP2 inhibition has been reported to partially rescue memory deficits in transgenic mouse models of diabetes and AD (Soeda et al., 2010; Kam et al., 2016) and to prevent metastasis in breast cancer cells (Ghosh et al., 2018). Since both SHIP1 and SHIP2 play critical roles in antagonizing microglial proliferation and phagocytosis, the use of both SHIP1 and SHIP2 inhibitors has been proposed in AD to enhance basal microglial homeostatic functions for therapeutic purposes (Pedicone et al., 2020). Although SHIP2 could be a potential biomarker and a valuable therapeutic target for AD, it remains largely elusive whether SHIP2 undergoes a significant alteration in subcellular localization and post-translational modifications during the progression of the disease. SHIP2 has more than 20 putative phosphorylation sites and its phosphatase activity and substrate recognition are, at least partially, regulated by phosphorylation, protein-protein interaction and subcellular localization (Elong Edimo et al., 2011). Given that SHIP2 is translocated to plasma membranes upon Aß-FcγRIIb interaction (Kam et al., 2016), subcellular localization of SHIP2 should be significantly altered in AD brains. Since FcγRIIb activation leads to tyrosine phosphorylation of SHIP2 (Muraille et al., 1999), the post-translational modifications of SHIP2 could be altered in the affected areas of AD brains. It remains to be carefully determined in *post-mortem* brain tissues of AD patients whether there are changes in SHIP2 subcellular localizations, post-translational modifications and the impact of SHIP2 upregulation in AD on PI amounts, particularly PI(3,4,5)P3 and PI(3,4)P2.

### SYNJ1 and SYNJ2

*SYNJ1* and *SYNJ2* are both highly conserved and their genetic variants are associated with cognitive abilities in a cohort with a mean age of 70 (Lopez et al., 2012). SYNJ1 is a brain-enriched presynaptic phosphatase involved in synaptic vesicle recycling, clathrin-coated vesicle uncoating at synapse (Cremona et al., 1999) and autophagosomal maturation within presynaptic terminals (Vanhauwaert et al., 2017). SYNJ1, whose gene is located in chromosome 21, is linked to endolysosomal abnormalities in Down syndrome (Cossec et al., 2012). Several mutations in *SYNJ1* gene are associated with early-onset Parkinsonism (Tran et al., 2020). Some of the polymorphisms in *SYNJ1* are also linked with age of onset in familial AD, late-onset AD and Down syndrome with AD (Miranda et al., 2018). SYNJ1 is expressed in neurons and is implicated in Aß toxicity (Berman et al., 2008), synaptic toxicity (McIntire et al., 2012) and Aß clearance (Zhu et al., 2013). The mRNA level of *SYNJ1* is significantly upregulated in *post-mortem* AD brains in association with *APOE* genotype (Zhu et al., 2015; Ando et al., 2020). SYNJ1 protein undergoes a significant solubility change and is co-enriched with PHF-tau in the sarkosyl-insoluble fraction (Ando et al., 2020). SYNJ1 immunoreactivity is detected in actin-positive Hirano bodies, some NFTs and plaque-associated dystrophic neurites in *post-mortem* human AD brains (Ando et al., 2020). Such aberrant alteration of mRNA levels, protein localization, and protein solubility of SYNJ1 could be applied to establish a valid biomarker for AD. While SYNJ1 is brain specific, its paralog SYNJ2 is ubiquitously expressed, but is also abundantly expressed in the synapse. In the temporal cortex from patients with depressive disorder, *SYNJ2* transcript expression is significantly decreased (Aston et al., 2005). Furthermore, differential methylation in the gene of *SYNJ2* has been also reported in association with aging in neuronal cells (Gasparoni et al., 2018).

### Potential Involvements of Other PI 5-Phosphatases in AD

The implication of the other members of PI 5-phosphatase family in AD remains largely unknown. Given that AD is associated with autophagic-endosomal-lysosomal dysfunction (Nixon et al., 2008), we speculate that INPP5E and OCRL, highly expressed in the brain and critical in autophagosome-lysosome fusion (De Leo et al., 2016; Hasegawa et al., 2016), might be involved in dysregulation of autophagy in AD brains.

### PI Metabolism and Autophagic-Endosomal-Lysosomal Abnormalities

Consistent with alterations of some PI 5-phosphatases observed in AD brains, there are substantial findings suggesting that PIs undergo dysregulation during the disease progression in AD brains (Stokes and Hawthorne, 1987) and in the AD blood plasma (Mapstone et al., 2014). In the AD prefrontal cortex where both amyloid and tau pathologies are abundant, the amounts of PI 3-phosphate (PI3P) and PI(4,5)P2 are significantly decreased (Morel et al., 2013). Deficiency of PIs in AD brains may be linked to autophagic-endosomal-lysosomal abnormalities observed in neurons of the AD patients even at an early stage (Nixon et al., 2008). Considering that PIs regulate membrane dynamics, we hypothesize that autophagic-endosomal-lysosomal abnormalities could be a potential target for developing AD biomarkers. For instance, endosomal morphology alteration has been observed in iPSC-neurons derived from AD fibroblasts (Israel et al., 2012) and AD blood monocytes (Corlier et al., 2015). Whereas the precise mechanisms underlying endosomal abnormalities remain to be determined, such endosomal alterations in peripheral cells could be considered as a novel potential approach to develop AD biomarkers.

## Discussion

Upregulation of some PI 5-phosphatases and PI dysregulations have been evidenced in AD and such alterations could be useful to develop new biomarkers for AD. Careful investigations will be needed to assess if these alterations are AD-specific or also associated with other diseases. Blood-based analyses of some PI 5-phosphatases, PI metabolism, transcriptomic and epigenetic changes have demonstrated alterations in AD and are conceivable strategies toward development of new biomarkers. Further studies will also be needed to evaluate the sensitivity and the specificity of these alterations during the progression of AD compared to currently available other markers such as those of PET and CSF analyses. These studies will be critical for deciphering the most reliable biomarkers and their complementarity for the diagnosis and the prognosis of this devastating disease.

## Author Contributions

All the coauthors participated in constructing the concept and writing the manuscript. KA, CE, and J-PB contributed conception and design of this article. All authors contributed to manuscript revision, read, and approved the submitted version.

## Conflict of Interest

The authors declare that the research was conducted in the absence of any commercial or financial relationships that could be construed as a potential conflict of interest.
